# Disaster risk management and SME resilience in low- and middle-income countries: A systematic review and evidence map

**DOI:** 10.4102/jamba.v18i1.2023

**Published:** 2026-05-07

**Authors:** Nomthandazo B. Mtetwa, Zamanguni F. Kubheka

**Affiliations:** 1 School of Commerce, College of Law and Management, University of KwaZulu-Natal, Durban, South Africa

**Keywords:** disaster risk reduction, business continuity, SME-resilience, enterprise risk management, evidence map

## Abstract

Small and medium-sized enterprises (SMEs) play an important role in employment creation and local economic stability, yet they are highly vulnerable to disasters and operational disruptions. This study synthesises existing evidence on risk management frameworks used by SMEs in low- and middle-income countries and examines how these approaches contribute to disaster risk reduction and business continuity. A PRISMA-guided systematic review was conducted using major academic databases and selected grey literature covering the period 2000 to 2025. Eligible studies examined the application or adaptation of structured risk management approaches, including enterprise risk management, business continuity management and related frameworks in SME contexts. The search identified 2 640 records, of which 76 studies met the inclusion criteria. The findings indicate that simplified versions of internationally recognised frameworks, particularly ISO-aligned risk management, COSO-based enterprise risk management and business continuity toolkits, are most frequently adopted by SMEs. Across sectors and regions, these approaches are associated with improved preparedness, increased risk awareness and shorter recovery times following disruptions. However, evidence linking adoption to sustained financial performance or long-term resilience remains limited. The review also highlights important gaps in the evidence base, including limited use of causal research designs, reliance on self-reported measures and the underrepresentation of micro, informal and women-owned enterprises.

**Contribution:**

Despite these limitations, the findings suggest that proportionate and context-sensitive practices can strengthen SME preparedness and recovery capacity. Policymakers and support institutions should promote accessible guidance, phased adoption pathways and practical continuity tools to better integrate SME resilience into local disaster risk reduction strategies.

## Introduction

Small and medium-sized enterprises (SMEs) play a key role in economic growth and job creation, although they remain highly vulnerable to disasters and disruptions (Organisation for Economic Co-operation and Development [OECD] 2020; United Nations Office for Disaster Risk Reduction [UNDRR] 2019). In many low- and middle-income countries (LMICs), informal business structures and limited resources make SMEs especially exposed to floods, pandemics, cyber incidents and supply chain breakdowns (Ali, Hanafiah & Mogindol 2023; Asgary, Ozdemir & Özyürek 2020; Auzzir, Haigh & Amaratunga 2018; Banwo et al. 2017). These overlapping risks highlight the need for formulating structured risk-management frameworks that strengthen preparedness, response and recovery (De Araújo Lima, Crema & Verbano 2020; United Nations 2015). While large organisations often apply formal risk-management systems, most SMEs in LMICs lack the finance, institutional support, and awareness needed to do the same (Kato & Charoenrat 2018; Sahebjamnia, Torabi & Mansouri 2015).

Therefore, many SMEs face long periods of downtime and slow recovery, affecting jobs and local economies (Asgary et al. 2020). Although global guidelines such as the Sendai Framework and International Organization for Standardization (ISO) 31000 promote business continuity planning, their use among SMEs in developing contexts remains inconsistent (ISO 2018; UNDRR 2019). This review bridges that key gap by analysing existing frameworks, assessing their quality and mapping how they contribute to SME-resilience and disaster-risk-reduction outcomes. By linking SME risk management with disaster risk reduction (DRR) principles, the present review aims to inform policy and practice for stronger, more adaptable businesses across multiple hazards (Ali et al. 2023; Asgary et al. 2020; Auzzir et al. 2018).

### Review objectives

The main aim of this review is to identify and evaluate existing SME risk-management frameworks that support business continuity and disaster resilience in LMIC. It also examines how these frameworks help SMEs prepare for, respond to and recover from different types of hazards (De Araújo Lima et al. 2020; Kato & Charoenrat 2018).

Primary outcomes of interest include indicators of business continuity and operational resilience, such as downtime duration (hours or days of disruption), time to restore operations, and preparedness or resilience scores derived from validated assessment tools (Sahebjamnia et al. 2015). Secondary outcomes include broader performance and compliance metrics, such as regulatory adherence, financial sustainability and integration of risk management into strategic planning (Ali et al. 2023; Asgary et al. 2020).

The review focuses on the following five main objectives:

- To identify and describe different types of risk management frameworks used by SMEs in developing countries.
- To examine how these frameworks are adapted to address specific challenges such as weak institutions, informality, financial limitations, disasters and currency risks.
- To assess the outcomes associated with these frameworks, including risk reduction, business continuity, resilience, performance, and compliance, and to explore how these outcomes are measured.
- To explore the barriers and enablers influencing the adoption of risk management frameworks, focusing on leadership, organisational culture, skills, technology and policy support.
- To highlight the existing gaps in the literature and identify priorities for future research and policy development related to SME risk management in developing contexts.

### Background

Small and medium-sized enterprises are vital to economic growth, job creation and poverty reduction, especially in developing regions (OECD 2020). However, in many LMICs, they operate in informal economies with limited access to finance, weak regulation, and poor institutional support (Kato & Charoenrat 2018). These challenges make SMEs highly vulnerable to hazards like floods, pandemics, and infrastructure failures, which disrupt recovery and weaken resilience. From a DRR perspective, SMEs are both vulnerable and essential for community recovery (Auzzir et al. 2018; UNDRR 2019). Their lack of preparedness and recovery planning often leads to severe business interruptions, affecting local supply chains, jobs and municipal recovery efforts (Ali et al. 2023; Asgary et al. 2020). Therefore, strengthening SME risk management not only poses a business issue but also forms a part of building local resilience and protecting livelihoods.

Despite the central role of SMEs in local recovery, no consolidated and DRR-oriented synthesis maps and risk-management frameworks are used in LMIC SME settings, nor are they adapted to resource constraints and informality, nor is there any evidence on continuity-related outcomes. This leaves policymakers and support agencies without a clear basis for designing proportionate guidance, incentives and capacity-building that can reduce downtime and improve recovery after disruption.

Many SMEs remain informal, uninsured and disconnected from disaster-management systems (Ali et al. 2023; Asgary et al. 2020). This challenge limits municipalities’ ability to integrate SMEs into hazard and continuity planning. Building SME capacity through training, ISO 31000 guidelines, and local business continuity models can bridge these gaps and improve coordination between enterprises and local authorities (ISO 2018; United Nations 2015). This review applies a DRR lens to explore how SME risk-management frameworks address hazard exposure, preparedness and continuity in LMICs. It maps existing approaches, highlights key gaps and provides insights to align SME practices with local disaster-management goals, thus supporting stronger and more coordinated resilience strategies.

### Evidence gap

Although risk-management frameworks for SMEs are widely discussed, existing research is fragmented and seldom linked to DRR principles (Ali et al. 2023; Asgary et al. 2020; De Araújo Lima et al. 2020). Most studies focus on financial resilience or operational continuity without connecting these outcomes to hazard exposure, preparedness, or community recovery (Sahebjamnia et al. 2015). Resultantly, evidence on how SME risk-management frameworks contribute to broader resilience strategies, especially in LMICs, remains limited.

Three main gaps stand out. First, the existing research lacks methodological consistency, with studies drawing on varied frameworks such as enterprise risk management (ERM), business continuity management (BCM), supply-chain resilience and cyber-risk governance (Auzzir et al. 2018). Some reviews have compared these approaches to identify common or differing indicators and outcomes. Second, a strong regional imbalance exists. The majority of the studies focus on formal SMEs in high-income countries, while evidence from LMICs is sparse and descriptive (Kato & Charoenrat 2018; OECD 2020). This research gap limits proper understanding of how SMEs in informal economies manage overlapping economic, environmental and technological risks.

Third, few studies measure DRR outcomes, such as preparedness, downtime reduction, or recovery speed (De Araújo Lima et al. 2020; UNDRR 2019). Many studies rely only on financial indicators, overlooking the multi-dimensional nature of business resilience. To address these gaps, the current review develops a systematic evidence map to consolidate existing studies, highlight dominant frameworks and identify research priorities. The evidence map visually summarises frameworks, geographic focus, methodological quality, and outcomes, thereby helping policymakers and researchers strengthen integrated SME-resilience planning (Miake-Lye et al. 2016; Haddaway et al. 2016). This aligns with Jàmbá’s focus on translating disaster-risk research into actionable, evidence-based policy for sustainable development.

## Research methods and design

### Methods (systematic desktop review)

This review is guided by the Preferred Reporting Items for Systematic Reviews and Meta-Analyses (PRISMA) 2020 reporting standards and follows established good practices for evidence-based management reviews. A protocol was registered on an open repository (e.g. Open Science Framework platform or OSF) prior to commencing the review, outlining the research questions, inclusion criteria, data sources and analysis plan to ensure transparency and reproducibility (Page et al. 2021; Tranfield, Denyer & Smart 2003). To support transparency, the search date, databases, screening steps, appraisal tools and synthesis approach were reported in a sequence aligned to PRISMA 2020.

### Eligibility criteria

#### Inclusion criteria

To ensure a focused and rigorous selection of relevant literature, the review was guided by the following inclusion criteria:

- Studies explicitly applying or developing a risk-management framework relevant to SMEs:
- Empirical or conceptual papers addressing preparedness, response, recovery, or continuity outcomes.
- Publications between 2000 and 2025 in English.
- Peer-reviewed journal articles, conference papers, theses, and verified grey literature (reports, guidelines and working papers).

#### Exclusion criteria

Studies were excluded where they fell outside the scope of the review or did not meet the specified methodological and contextual criteria, as outlined below:

- Studies focusing exclusively on large corporations or public institutions.
- Papers lacking explicit RM (risk-management)/BCM/ERM/SCRM (supply chain risk management)/Cyber-RM frameworks.
- Commentaries, editorials, and opinion pieces without primary or secondary data.
- Non-English studies where full translation was not possible.

Grey-literature sources were screened using the same eligibility parameters and evaluated for credibility, completeness and citation provenance.

### Information sources

An extensive search was performed across major multidisciplinary and specialist databases commonly used in management research, including Scopus, Web of Science, *Elton B. Stephens Company (EBSCO host research databases)* Business Source, ProQuest, IEEE Xplore (for cyber-risk tools), and EconLit. To reduce publication bias, grey literature was also screened from organisations such as the World Bank, International Finance Corporation (IFC), OECD, United Nations Industrial Development Organization (UNIDO), International Labour Organization (ILO), UNDRR, African Development Bank (AfDB), Asian Development Bank (ADB), national SME agencies, central banks and standards bodies. The search was complemented with Google Scholar (top ~200 results by relevance), citation tracking from key studies, and manual search of relevant special issues (Tranfield et al. 2003).

### Search strategy

Both controlled vocabulary and free-text terms were combined to capture relevant studies on SMEs, RM frameworks, and developing-country contexts, with search strings adjusted for each database. The main Boolean structure included the following:

- (‘small and medium enterprise’ OR SME OR SMME OR MSME) AND
- (‘risk management’ OR ‘enterprise risk management’ OR ERM OR ‘ISO 31000’ OR ‘COSO’ OR ‘business continuity’ OR ‘resilience’ OR ‘risk maturity’ OR ‘supply chain risk’) AND
- (developing OR ‘low income’ OR ‘lower middle income’ OR Africa OR Asia OR ‘Latin America’ OR country-specific terms).

As qualitative and mixed-method studies are common in LMIC SME research, the SPIDER tool (Sample, Phenomenon of Interest, Design, Evaluation, Research type) was also used to guide supplementary searches (Cooke, Smith & Booth 2012). Full database-specific search, necessary strings are provided in Appendix 1.

### Study selection

All records were imported into a reference manager, and duplicate studies and/or data were removed. Screening took place in two stages: In the first stage, titles and abstracts, and in the second stage, full texts were screened, using the defined eligibility criteria. As this was a desktop review, a single-reviewer process was followed, with a 10% – 20% reliability check by a second reviewer on a random sample; any differences were discussed to ensure consistency. Reasons for excluding studies at the full-text stage were documented, and a PRISMA flow diagram is provided in the Appendix/Results (Page et al. 2021). The screening form is available in Appendix 2 (Page et al. 2021; Tranfield et al. 2003).

### Data extraction

A standardised extraction form was used (Appendix 3), with 10% of entries double-checked for accuracy. Data fields captured included the following:

- Bibliographic details (year, country/region, sector), SME definition/size, and informality indicators.
- Type of RM framework (ISO 31000, Committee of Sponsoring Organizations [COSO], BCM, SCRM, cyber, finance/Insurtech) and maturity level.
- Implementation aspects (tools, processes, training and digital supports).
- Contextual factors (e.g. institutional quality, financing, disaster risks, corruption, infrastructure, connectivity, owner characteristics).
- Outcomes and metrics (quantitative indicators, qualitative themes).
- Study design and appraisal results.
- Key findings, barriers/enablers, and recommendations.

For qualitative studies, thematic codes and broader themes were developed using thematic synthesis methods (Thomas & Harden 2008).

### Synthesis approach

This review employed narrative synthesis because of the heterogeneity of designs and outcomes, consistent with established approaches to synthesising complex and diverse evidence (Ogilvie et al. 2008). Findings were grouped by framework family (ERM, BCM, SCRM, Cyber-RM) and mapped to mechanisms (e.g. leadership, learning, finance, digital controls) and outcomes (continuity, downtime and resilience). Where quantitative comparisons were feasible, vote-counting of effect direction (positive/neutral/negative) was applied, complemented by harvest plots to visualise frequency and quality distribution (eds. Higgins et al. 2020). Heterogeneity was addressed descriptively by sector, region and study design; no meta-analysis was conducted due to incompatible metrics.

### Analytical framing (context–intervention–mechanism–outcome/sample, phenomenon of interest, design, evaluation, research)

To maintain a problem-oriented synthesis, the review applied CIMO (Context–Intervention–Mechanism–Outcome) logic (Denyer, Tranfield & Van Aken 2008) and SPIDER for qualitative evidence (Cooke et al. 2012):

- Context (C): SMEs in developing economies (income categories noted per study).
- Intervention (I): RM frameworks, practices, tools and programmes (ERM, ISO 31000, BCM/DRR, SCRM, cyber, maturity models, fintech/insurtech).
- Mechanism (M): Organisational capabilities, governance, culture, and learning processes enabling RM.
- Outcomes (O): Risk exposure, continuity, resilience, performance, compliance and survival.

Findings are mapped to CIMO elements in evidence tables and the narrative to make causal assumptions explicit (Cooke et al. 2012; Denyer et al. 2008).

### Bias and limitations management

Publication bias was reduced by searching multiple databases and including high-quality grey literature. However, language bias is encountered due to English-only inclusion. The single-reviewer workflow was mitigated through calibration and second reviewer checks on a subset. Anticipated heterogeneity in designs and measures was addressed through transparent reporting, sensitivity analysis and evidence mapping. A reflexivity statement acknowledges the reviewers’ disciplinary backgrounds and potential interpretive biases (Page et al. 2021).

## Literature review

Research on risk management in SMEs has evolved through multiple theoretical perspectives that explain how firms anticipate, absorb and recover from disruptions. Four major framework families dominate the literature: (1) ERM, (2) BCM, (3) SCRM, and (4) Cyber-risk and digital resilience frameworks (Auzzir et al. 2018; ISO 2018; Sahebjamnia et al. 2015). Each framework provides a distinct but complementary approach to managing uncertainty and building adaptive capacity (Yang et al. 2018; Auzzir et al. 2018; ISO 2018; Sahebjamnia et al. 2015).

Enterprise risk management frameworks emphasise holistic identification and mitigation of strategic, financial and operational risks (COSO 2017). In contrast, BCM frameworks focus on the continuity of critical functions before, during and after crises, aligning closely with disaster preparedness and response cycles (Herbane 2019). Supply chain risk management models extend this thinking to inter-organisational networks, highlighting coordination across suppliers, distributors, and logistics systems that are often lifelines for SMEs (Ivanov & Dolgui 2020). Finally, cyber-risk frameworks foreground information security, data protection and digital business continuity issues that have become increasingly relevant with the rise of e-commerce and remote operations (Hong et al. 2019; Ali et al. 2023; Kabanda, Tanner & Kent 2018).

Across these families, resilience emerges through four recurrent mechanisms: Leadership, learning, finance and digital controls. Firstly, leadership shapes organisational culture and risk awareness, influencing how SMEs prioritise preparedness and allocate resources (Kato & Charoenrat 2018). Secondly, learning mechanisms, including training, knowledge sharing and post-event reflection, enable adaptive behaviour and incremental improvement (Duchek 2020). Thirdly, financial mechanisms such as savings, credit lines and insurance determine an enterprise’s capacity to absorb shocks and sustain recovery (OECD 2020). Lastly, digital controls provide real-time monitoring, redundancy, and communication systems that reduce downtime and preserve continuity (Ali et al. 2023; Asgary et al. 2020).

Collectively, these mechanisms operate as mediators between the adoption of a framework and observable outcomes such as reduced downtime, quicker restoration of operations and improved resilience scores. The conceptual relationship between these elements is illustrated in Appendix 1 Figure 1-A1, which synthesises insights from multiple studies and establishes an analytical foundation for this review.

### Theoretical and conceptual framework

#### Conceptual framework

Building on recent work on organisational resilience and SME risk management, the review uses a capability-oriented lens to explain why some frameworks are more practicable for SMEs in LMICs than others. Resilience can be understood as a set of capabilities that enable businesses to anticipate, cope and adapt during disruption, rather than as a single attribute or checklist (Duchek 2020). In parallel, systematic reviews of SME risk management emphasise that proportionate routines and learning mechanisms matter more than formal compliance when resources are limited (Ali et al. 2023; De Araújo Lima et al. 2020).

Accordingly, the conceptual framework links four framework families (ERM, BCM, SCRM and cyber-risk approaches) to continuity outcomes through four mediating capability domains: (1) leadership and governance, (2) learning and routines, (3) financial slack and access to recovery finance and (4) digital controls that support visibility and redundancy. These domains are mutually reinforcing. For example, supply-chain continuity depends not only on supplier diversification but also on decision rights and timely information flows, which became especially visible during COVID-19-related disruptions (Ivanov & Dolgui 2020).

The framework is operationalised in the synthesis through CIMO logic: The LMIC SME context shapes which interventions are feasible; interventions trigger mechanisms (capability domains) that in turn influence outcomes such as reduced downtime, faster restoration and improved preparedness. This framing supports the evidence map by making assumptions explicit and by helping to interpret mixed results across regions, sectors and study designs.

#### Theoretical and analytical foundations

To understand how SMEs in LMICs take up and adjust RM frameworks, several management theories provide useful perspectives. Contingency theory argues that practices must fit external conditions, meaning SMEs in unstable environments adapt their frameworks to realities like weak regulations or frequent disasters (Rehman et al. 2020). Dynamic capabilities theory focuses on how firms sense, seize and adapt to change (Teece 2007), which is valuable for explaining how SMEs build flexible RM practices under resource constraints.

For this review, the CIMO framework is used as the guiding structure. The context is SMEs in developing countries, where challenges are heightened by weak institutions and limited resources. The intervention refers to RM frameworks, practices and tools, whether adapted from ISO/COSO models or sector-specific approaches like SCRM or business continuity planning. The mechanisms include factors such as organisational skills, governance, leadership and learning processes that influence how RM is adopted and applied. The outcomes cover reduced risk exposure, stronger continuity and resilience, better performance, improved compliance and survival of the business (De Araújo Lima et al. 2020). This framework provides the basis for the analysis and shows why SME risk management in LMICs must be studied at multiple levels, linking governance systems with organisational capacities and broader institutional conditions.

### Ethical considerations

This article followed all ethical standards for research without direct contact with human or animal subjects.

## Results

### Study selection

After searching sources published between January 2000 and August 2025, the search process identified 2412 records from major databases (Scopus, Web of Science, EBSCO, ProQuest, IEEE Xplore, EconLit) and a further 228 from supplementary sources such as Google Scholar, backwards and forward citation chasing, and institutional repositories. After removing 612 duplicates, 2028 titles and abstracts were screened, of which 1706 were excluded because they were out of scope, did not focus on SMEs or LMICs, or were conceptual papers without clear methods. This left 322 full-text articles to be assessed for eligibility. Of these, 246 articles were excluded, most commonly because they did not focus on LMICs (*n* = 104), did not address SMEs or did not provide an SME-specific sub-analysis (*n* = 62), lacked sufficient methodological detail (*n* = 35) or were unrelated to organisational RM (*n* = 45).

In total, 76 articles met the study inclusion criteria and were included in the synthesis: 61 peer-reviewed journal articles and 15 pieces of high-quality grey literature. Figure 1 summarises the selection process, illustrating different stages of identification, screening, eligibility and inclusion. The most common reasons for exclusion were: (1) studies focused solely on large corporations, (2) lack of an explicit risk-management framework, or (3) outcomes unrelated to preparedness, continuity or recovery. This systematic approach ensures methodological transparency and replicability, consistent with PRISMA 2020 guidance (Page et al. 2021).

**FIGURE 1:**
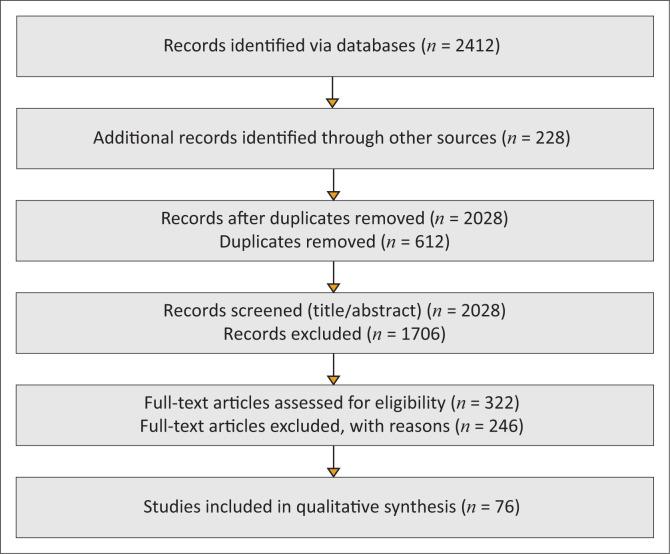
Preferred Reporting Items for Systematic Reviews and Meta-Analyses 2020 flow diagram.

### Characteristics of included studies

Table 1 provides a summary of the 76 included studies, categorised by region, framework family, methodological design, and quality appraisal tier. The majority of the studies (68%) employed mixed or qualitative designs, whilst 32% applied quantitative or modelling approaches. Geographically, the evidence base remains skewed towards high-income or upper-middle-income countries, with Asia (35%) and Europe (28%) dominating. Only 22% of studies originate from Africa and 10% from Latin America. This regional imbalance underscores the need for greater LMIC representation in SME-resilience research (Ali et al. 2023; Asgary et al. 2020). Framework distribution shows ERM (25%), BCM (30%), SCRM (20%), and Cyber-Risk frameworks (15%), with hybrid or multi-framework models comprising the remaining 10%. Across regions, BCM approaches were most common in Asia and Europe, while ERM and SCRM approaches featured prominently in manufacturing and logistics sectors. In this review, ‘sector’ is used to denote broad areas of economic activity (e.g. manufacturing, services), whilst ‘industry’ refers to more specific segments within a sector (e.g. food manufacturing).

**TABLE 1:** Counts summary.

Stages of review process	Records (*n*)
Records identified via databases	2412
Additional records identified through other sources	228
Duplicates removed	612
Records after duplicates removed	2028
Records screened (title/abstract)	2028
Records excluded at title/abstract	1706
Full-text articles assessed for eligibility	322
Full-text articles excluded (with reasons)	246
Studies included in qualitative synthesis	76

### Quality appraisal summary

Quality assessment using the Mixed Methods Appraisal Tool (MMAT) and Critical Appraisal Skills Programme (CASP) tools, alongside established guidance for appraising prevalence and observational studies (Barker et al. 2023), indicated that 42% of studies were rated high, 38% moderate, and 20% low quality. Common weaknesses included insufficient triangulation, lack of DRR-aligned indicators and poor external validity. Few studies explicitly reported ethical review or data-sharing transparency, reflecting a need for stronger methodological rigour across SME-resilience research (De Araújo Lima et al. 2020). Quality assessment using tools such as MMAT and the Critical Appraisal Skills Programme (CASP 2022).

### Evidence synthesis

**FIGURE 2-A1:**
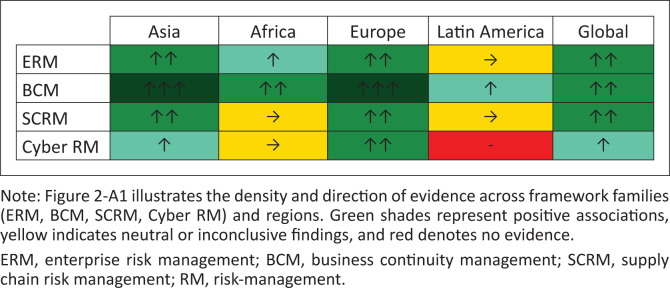
Evidence – Map heat matrix: framework × families × regions × outcomes.

To visualise distribution of evidence across framework families, regions, and outcomes, an evidence-map heat matrix (Appendix 1 Figure 2-A1) was developed drawing on bibliometric and mapping approaches commonly used to visualise research landscapes (Van Eck & Waltman 2010). The colour intensity represents the density of evidence, whilst the directional arrows (↑ = positive, → = neutral, = no evidence) indicate the reported effect of each framework on continuity, downtime reduction and resilience outcomes. The map shows that BCM frameworks dominate the literature, especially in Asia and Europe, where evidence for positive continuity outcomes is strongest. By contrast, Cyber-Risk Management (Cyber RM) frameworks remain under-represented, with only a few studies assessing digital resilience in LMIC contexts. Supply chain risk management shows moderate global coverage, largely focused on manufacturing and logistics sectors. Overall, the evidence synthesis reveals a significant imbalance, with limited African and Latin-American representation and a few studies assessing preparedness or recovery as explicit DRR outcomes.

### Gaps and inconsistencies

Four thematic insights emerge from the evidence synthesis. Firstly, leadership and organisational learning consistently underpin successful implementation across all frameworks. Secondly, financial capacity remains a limiting factor for SME preparedness and recovery, particularly in LMICs. Thirdly, digital controls and cybersecurity are gaining prominence, though they lack systematic integration into traditional RM frameworks. Lastly, contextual adaptation – the tailoring of global frameworks to local hazards and institutional realities – is weak, calling for localisation strategies in future DRR-aligned SME research (OECD 2020; UNDRR 2019).

## Discussion

### Interpretation

The findings suggest that SME risk management in LMICs is best understood as pragmatic and incremental. Small and medium-sized enterprises cannot fully adopt formal ISO or COSO models due to limited resources, but instead they adapt simplified practices such as short risk registers, continuity drills and basic cyber hygiene (Brustbauer 2016; De Araújo Lima et al. 2020). Thai SMEs, for instance, often show partial preparedness without full documentation (Kato & Charoenrat 2018). Turkish SMEs report strong awareness of risks but weaker formalisation (Asgary et al. 2020). South African SMEs face growing cyber risks, but adoption is constrained by high cost and inadequate leadership support (Kabanda et al. 2018).

### Alignment with international standards

Although global standards such as ISO 31000 and COSO ERM provide valuable principles, SMEs in LMICs only partially align with them. Leadership commitment and basic monitoring practices are present in some cases, particularly export-oriented SMEs tied into international supply chains (Thun, Drüke & Hoenig 2011). Divergence is most visible in governance structures, documentation, and systematic reporting. These are often unrealistic for SMEs. Instead, staged adoption pathways starting with simple tools and moving towards more integrated practices appear most feasible (Bromiley et al. 2015; Oliva 2016).

### Policy and ecosystem roles

External support is critical for scaling SME RM. Financial instruments such as credit guarantees and index-based insurance can reduce barriers while bundling RM training (Ali et al. 2023). Small and medium-sized enterprises agencies and associations can help diffuse simplified templates and provide peer-learning opportunities (Kato & Charoenrat 2018; Thun et al. 2011). Municipal involvement in disaster preparedness and recovery, as evident in Thailand and Tanzania, accelerates SME recovery (Pathak & Ahmad 2016; Sakijege 2024). Digital public goods such as open-source RM tools or cyber hygiene modules can also lower costs and spread adoption (Kabanda et al. 2018).

### Research implications

Future research should address key gaps: Conduct longitudinal and quasi-experimental studies, standardise outcome measures, evaluate cost-effectiveness of RM interventions, test integrated frameworks covering multiple risk domains and expand coverage to informal and women-owned SMEs (Ali et al. 2023; De Araújo Lima et al. 2020; Kabanda et al. 2018; Sakijege 2024; Thun et al. 2011).

### Synthesis

In sum, SMEs in developing economies demonstrate resilience through context-appropriate and proportionate RM practices rather than wholesale adoption of international standards. International Organization for Standardization 31000 and COSO ERM remain important benchmarks, but their application in LMICs requires tailoring to local realities. Policy ecosystems and digital innovations can enable adoption, whilst research must close evidence gaps to better guide practice. Ultimately, resilience in SMEs is achieved not through replication of corporate RM systems but through adaptive, dynamic capabilities suited to volatile environments (Bromiley et al. 2015; Oliva 2016; Teece 2007).

### Limitations of the current review

#### Coverage and language constraints

This review aimed to capture a wide range of literature by drawing on multiple academic databases and selected grey literature sources. Despite these efforts, the overall coverage cannot be considered fully exhaustive. Indexing practices differ across platforms, which means that some relevant material, especially that appearing in less prominent regional or practice-based outlets, may not have been retrieved. Furthermore, this review focused only on English-language studies, which creates the potential for language-related bias. Work published in other widely spoken languages may contain valuable perspectives that are under-represented here, particularly those from regions where English is not the dominant academic language. This limitation may result in an overemphasis on evidence generated in anglophone contexts.

#### Heterogeneity precluding meta-analysis

The pool of included studies in this review is highly diverse in terms of methodology, geographic focus, sectoral coverage, and the method of outcome measurement. Different frameworks, industries and regions adopt unique approaches. This causes inconsistency in how key concepts are defined and operationalised. Therefore, the studies were too heterogeneous to allow for the application of formal meta-analytic techniques. Instead, the findings had to be presented narratively and through broad evidence mapping. Another limitation is that, despite a valid approach, this narrative synthesis carries a higher degree of subjectivity compared to more statistical techniques.

#### Reliance on reported outcomes and self-reports

A significant proportion of the primary literature examined in this review relied heavily on self-reported outcomes. This reliance raises several concerns about accuracy, as responses may be influenced by factors such as social desirability, recall limitations or inconsistent interpretation of survey items. In cases where objective indicators were used, they were often applied inconsistently, lacked verification or showed variation in definition across studies. Consequently, the results and associations described in the included sources should be treated with caution, as they may not accurately reflect the realities being measured.

#### Limited causal inference

The study designs underpinning the available evidence were predominantly cross-sectional surveys and qualitative case studies. Stronger methodological approaches, such as longitudinal or quasi-experimental designs, were rarely observed. Hence, the review is unable to establish strong causal claims about the relationship between the practices studied and the outcomes identified. Many of the associations reported may instead be influenced by unobserved factors or selection effects. These factors reduce the certainty with which conclusions can be drawn.

#### Small and medium-sized enterprises definition variability and external validity

The definition of SMEs is not consistent across the studies reviewed. Thresholds vary by jurisdiction and sometimes even by programme within a single country. Some studies include micro-enterprises while others do not, and many omit informal businesses altogether. Given the prevalence of informality in developing economies, this exclusion limits the external validity of the findings. The results may therefore be more applicable to formal, urban-based enterprises than to rural or informal operations.

#### Regional and sectoral imbalances

The geographic distribution of studies included in the review was uneven. Some regions, particularly middle-income or more industrialised countries, were represented more strongly, while others had little or no coverage. Similarly, some sectors, such as manufacturing and agriculture, were discussed in greater detail than services or retail. These imbalances may skew the overall synthesis towards certain contexts and away from others, potentially limiting the breadth of the conclusions.

#### Grey literature inclusion and appraisal challenges

The inclusion of grey literature was intended to broaden the evidence base and minimise publication bias. However, the methodological quality of grey literature varies substantially, and reporting is often less detailed than in peer-reviewed studies. Standard appraisal tools are not always well-suited to evaluating these types of documents, which can make assessment more difficult. As a result, the overall appraisal of evidence quality should be interpreted with this limitation in mind.

#### Screening and extraction limitations

The process of screening and extracting data for this review involved human judgement at several stages. While measures were taken to ensure consistency and reliability, such as calibration exercises and checks on subsets of data, the possibility of misclassification, oversight or incomplete data capture persists. This limitation is particularly relevant given the long timeframe of the review, which spanned several decades and included a wide variety of terminology and practices.

#### Framework classification and overlap

The classification of studies into distinct frameworks was complicated by the fact that many interventions combine multiple approaches. For example, some frameworks integrate elements of preparedness, continuity and risk management simultaneously. The effort to code studies into single categories may therefore obscure the complexity and interconnections that exist in practice.

#### Time sensitivity of findings

The issues addressed in this review are dynamic and subject to rapid change. New challenges, technological developments, and shifts in the broader environment may quickly render findings obsolete. While the review covered literature up until the present, its conclusions should be read with the understanding that the field continues to evolve.

#### Synthesis choices and residual bias

The way evidence was synthesised through mapping and narrative approaches was necessary but not without risk. Cells or categories that were better documented inevitably appear more prominent, while areas with little published evidence may appear less important than they actually are in practice. These synthesis choices could therefore unintentionally reinforce existing biases in the literature.

#### Implications

Taking these limitations into account, the findings of this review should be interpreted as indicative rather than definitive. They highlight trends and point towards practical insights but cannot be taken as precise measurements of effect. The limitations also emphasise the need for future research that adopts more robust designs, uses consistent definitions, incorporates under-represented regions and enterprise types and provides stronger verification of reported outcomes. Addressing these gaps would enhance both the reliability and the policy relevance of subsequent reviews.

This review suggests that effective strategies for SMEs in lower-resource environments are gradual, proportionate and adaptable to context, rather than complex, compliance-heavy frameworks. The most sustainable resilience outcomes appear when SMEs begin with foundational practices (basic registers, simple continuity exercises, minimum digital safeguards), embed risk awareness into daily routines (tracking suppliers, recording incidents, setting a few continuity indicators), and then scale up to more formalised, standards-aligned processes as resources and organisational maturity increase. Success consistently depends on practical mechanisms: Clear leadership signals, streamlined routines, accessible templates and iterative learning that builds from real events.

The effectiveness of resilience strategies is not uniform, but varies according to the nature of the sector and the specific contextual conditions within which organisations operate:

- In production- and supply-focused sectors, practices that strengthen supply-chain resilience, such as diversification of sources, maintaining safety stocks and flexible logistics, tend to produce the most significant improvements in continuity.
- In urban service industries and digitally dependent SMEs, low-cost digital security measures such as better password management, multifactor authentication, regular backups and controlled system access yield substantial returns.
- Small and medium-sized enterprises integrated into global or regional value chains adopt practices more rapidly when incentives and expectations are set by buyers or partners.

International frameworks such as ISO 31000 and COSO ERM continue to provide useful guidance, but their application must be scaled to SME realities. At earlier stages, insisting on extensive documentation or committee structures is impractical; more valuable are simple cycles of review, active maintenance of a risk register, and a few targeted exercises relevant to priority hazards. As organisations grow, they can refine their risk appetite statements, align identified risks with broader strategy, and adopt structured scenario analysis where it directly aids decision-making. In this way, capabilities develop before compliance becomes the main driver. The strongest evidence from across the literature highlights improvements in preparedness and recovery, such as faster restoration of operations and reduced disruption time. While more rigorous studies are needed to confirm direct causal impacts on profitability or long-term survival, the trends suggest that SMEs with embedded risk routines experience fewer severe interruptions. External conditions either amplify or limit these gains: Access to financial products that support recovery, diffusion of standards through sector agencies, and affordable digital tools lower barriers to adoption. Similarly, procurement policies that reward even minimal risk management maturity can encourage wide uptake with little additional burden. The implications are clear. Small and medium-sized enterprises owner–managers can take achievable incremental steps: Develop a short risk register, run a drill for one priority hazard, identify at least one alternative supplier and apply core digital safeguards; then revisit and refine these actions after disruptions and during routine reviews. Policymakers and intermediaries should prioritise accessible, sector-specific training, promote practical toolkits, embed recognition of risk-management practices in finance and procurement, and connect businesses with relevant hazard and recovery data. Researchers should strengthen the evidence base through longitudinal and standardised designs, and include diverse enterprise types, particularly informal and women-led businesses.

In conclusion, building resilience in SMEs in developing economies is less about replicating the bureaucratic processes of larger firms and more about nurturing adaptive, learning-based routines tailored to local risks and capacities. A phased, context-sensitive approach supported by enabling financial systems, practical standards and accessible technologies provides the most credible pathway to enduring continuity and, ultimately, stronger enterprise outcomes.

## Recommendations and managerial implications for stakeholders

### Managerial implications

For SME owner–managers, incremental steps are most practical: Develop a simple risk register, conduct scenario drills for common hazards, diversify suppliers, adopt affordable cyber controls, and track continuity metrics such as downtime and restoration time (Ali et al. 2023; Brustbauer 2016; Kabanda et al. 2018; Kato & Charoenrat 2018; Thun et al. 2011). These measures, while modest, strengthen resilience and prepare SMEs for scaling up RM sophistication as resources allow.

### Recommendations

Based on the evidence reviewed from LMIC contexts, effective SME risk management is best understood as proportionate, staged and context-sensitive, rather than as a rigid compliance exercise. The recommendations are grouped for SMEs themselves, policymakers and intermediaries, and the research community (Ali et al. 2023; De Araújo Lima et al. 2020).

### For small and medium-sized enterprises (practice roadmap)

In the longer term, SMEs can progress towards ‘lite’ ISO-aligned ERM by defining risk appetite in plain terms, conducting scenario analyses, digitising monitoring of risks and incidents and embedding continuous improvement cycles. These practices enable firms to approximate international standards in a proportionate way (Bromiley et al. 2015; Oliva 2016).

### For policymakers and intermediaries

Policymakers and ecosystem actors can play a catalytic role by lowering barriers and diffusing simplified RM practices. Key measures include subsidising sector-specific training delivered via chambers or Technical and Vocational Education and Training (TVET) colleges, publishing simplified toolkits and templates in local languages, and expanding access to credit guarantees or parametric insurance linked to basic RM documentation (Ali et al. 2023; Oliva 2016). Governments can also strengthen municipal hazard data and early warning systems, ensuring SMEs receive timely information on floods, outages or restoration schedules (Pathak & Ahmad 2016; Sakijege 2024). Procurement systems can reward suppliers with basic RM maturity, diffusing practices through value chains (Thun et al. 2011). Finally, support should target the ‘last mile’ of informal and micro-SMEs with mobile-first tools, waived fees and delivery through associations and women’s networks (Rehman et al. 2020; Sakijege 2024).

### For researchers

Several research priorities emerge. First, there is a need to develop standardised outcome measures (e.g. downtime, supplier stock-out days, cyber incident rates) and publish open datasets that enable cumulative evidence-building (Ali et al. 2023). Second, longitudinal and quasi-experimental designs are required to move beyond descriptive studies and provide stronger causal evidence on survival and performance impacts (De Araújo Lima et al. 2020). Third, cost-effectiveness analyses should be conducted to show the benefits of low-cost RM controls, guiding both policy and SME investment choices. Fourth, sector-specific RM playbooks should be co-designed and tested in areas such as agrifood, construction and retail (Thun et al. 2011). Fifth, implementation science approaches can be used to understand what works, where, and why in LMIC SME settings, particularly under informality (Rehman et al. 2020). Finally, future studies should integrate multiple risk domains – climate, supply chain, and cyber – while prioritising equity through oversampling of women-owned and informal SMEs (Kabanda et al. 2018; Sakijege 2024).

## Conclusion

In concluding this PRISMA-based systematic review and evidence map, the available evidence suggests that structured risk management and business continuity approaches can be both feasible and valuable for SMEs in LMICs, provided that they are scaled to local realities. Across the 76 included studies, the most consistent benefits are seen in improved preparedness, stronger risk awareness and shorter recovery times, rather than in sustained financial gains, which remain under-examined and difficult to attribute. The review also makes clear that resilience is highly context-dependent. Production and supply-oriented SMEs tend to benefit most from supply continuity measures, while service-based and digitally reliant businesses often gain quickly from basic cyber hygiene and low-cost digital safeguards. At the same time, the unevenness of the evidence base is striking: Micro and informal enterprises and women-owned businesses are under-represented, and robust causal designs remain an exception. These gaps matter because they limit confidence about which interventions work best, for whom and under which conditions.

Taken together, the findings support a phased, capability-building pathway in which SMEs begin with simple routines and practical tools, strengthen learning and leadership around risk and progressively move towards more formal, standards-aligned practices as capacity grows. For policy and practice, this finding indicates the value of accessible sector guidance, simplified metrics and incentives embedded in finance, procurement and local support systems. For research, it underlines the need for better outcome measurement, more comparative and longitudinal designs, and deliberate inclusion of neglected enterprise groups, so that the field can move from promising trends to stronger, decision-ready evidence.

### Study contribution

This study contributes to disaster risk studies by providing a consolidated, DRR-oriented synthesis of how risk management and business continuity frameworks are used and adapted by SMEs in LMICs. By applying PRISMA methods alongside an evidence map, the review moves beyond isolated case-based insights and offers a structured overview of what framework families dominate the literature, where the evidence is concentrated, and which outcomes are most consistently reported. The study also advances conceptual understanding by linking SME risk management to continuity and recovery mechanisms that matter in disaster contexts, while making visible the conditions under which particular approaches appear most practical. Importantly, the review highlights policy-relevant gaps and implementation challenges, including the limited inclusion of informal, micro and women-owned enterprises, the scarcity of causal designs and the uneven measurement of DRR outcomes, such as downtime and recovery speed. In doing so, the article provides a clearer foundation for proportionate guidance, support programmes and future research aimed at strengthening SME-resilience as part of broader community and local economic recovery.

## Figures and Tables

**FIGURE 1-A1:**
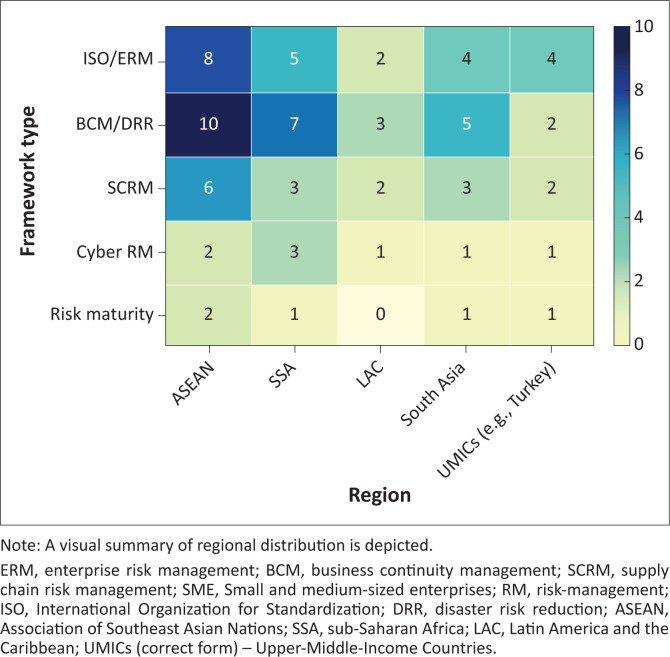
Evidence map – Density of small and medium-sized enterprises risk-management framework studies by region.

## Appendix 1

**TABLE 1-A1:** Study overview (illustrative subset of included low- and middle-income countries studies).

Region	Sector	Framework family	Design	Sample range	Quality tier	Dominant outcomes
Africa (*n* = 12)	Manufacturing, services	BCM, ERM	Qualitative	10–55 SMEs	Moderate	Preparedness, recovery
Asia (*n* = 27)	Manufacturing, ICT	BCM, ERM, SCRM	Mixed	20–300	High	Continuity, downtime
Europe (*n* = 21)	Finance, logistics	ERM, SCRM	Quantitative	30–400	High	Performance, compliance
Latin America (*n* = 8)	Retail, tourism	ERM	Qualitative	8–60	Low–moderate	Preparedness
Global/comparative (*n* = 8)	Multi-sector	Hybrid (ERM + Cyber RM)	Modelling	-	High	Resilience index

Note: The table summarises regional and sectoral distribution, framework families, designs, sample ranges, quality tiers, and dominant outcomes.

RM, risk-management; ERM, enterprise risk management; BCM, business continuity management; SCRM, supply chain risk management; ICT, Information and Communication Technology.

**TABLE 2-A1:** Frameworks, components and implementation level (illustrative subset).

Framework family	Core components	Typical SME practices (LMIC context)	Implementation level	Primary capability domain	Expected outcomes
Enterprise Risk Management (ERM) (e.g. ISO 31000, COSO)	Identification and assessment of risks, use of risk registers, governance and monitoring processes	Informal risk identification, owner-managed decision-making, basic prioritisation of risks, limited documentation	Partial and incremental adoption	Leadership and governance	Improved risk awareness and prioritisation, gradual integration into business strategy
Business Continuity Management (BCM)	Business impact analysis, continuity planning, recovery procedures and crisis response processes	Simplified continuity plans, backup arrangements, manual recovery processes and occasional drills	Moderate adoption in simplified form	Learning and organisational routines	Reduced downtime, faster recovery and improved preparedness
Supply Chain Risk Management (SCRM)	Supplier diversification, inventory management, logistics coordination and disruption monitoring	Use of alternative suppliers, safety stock practices, informal supplier networks and reactive adjustments	Moderate and context-dependent adoption	Operational coordination and learning	Improved supply continuity, reduced exposure to disruptions and enhanced resilience
Cyber-risk and digital resilience frameworks	Data protection, cybersecurity measures, system redundancy and digital continuity planning	Basic cybersecurity practices, limited investment in systems and reliance on external support	Low but emerging adoption	Digital controls and systems	Reduced vulnerability to cyber risks and improved continuity of digital operations
Hybrid or integrated frameworks	Combination of ERM, BCM and SCRM elements adapted to context-specific needs	Use of blended tools, iterative learning and simplified integrated approaches	Low to moderate, depending on context	Integrated capability domains (leadership, learning, finance and digital controls)	Improved preparedness, adaptability and overall resilience capacity

Note: Adapted and synthesised from the reviewed literature on SME risk management frameworks in LMIC contexts.

**TABLE 3-A1:** Outcomes and measurement (illustrative subset).

Study	Outcomes	Measurement approach
Kato and Charoenrat (2018)	BCM adoption/readiness; assistance needs	Survey items: presence of BCP, training, drills
Pathak and Ahmad (2016)	Recovery capacity; time to restore	Qualitative case tracing
Asgary et al. (2020)	Risk exposure profile (economic, geopolitical, tech, env., societal)	Likelihood and impact scales; risk values
Kabanda et al. (2018)	Cyber practices; barriers	Semi-structured interviews; cross-case themes
Sakijege (2024)	Continuity/recovery factors	Field observations; thematic analysis
Ali et al. (2023)	Evidence map of BCM outcomes	SLR coding of outcomes

Note: The table provides details on how resilience indicators were operationalised across selected studies.

BCM, business continuity management; BCP, Business Continuity Planning; SLR, Systematic Literature Review.

## Appendix 2: Screening Form (Study Selection Tool)

This form was used during title, abstract and full-text screening to ensure consistent application of the eligibility criteria.

**Table:**

Criterion	Yes	No	Unclear
Does the study focus on SMEs or include SME-specific analysis?	□	□	□
Is the study conducted in a low- or middle-income country?	□	□	□
Does the study address risk management, business continuity or resilience?	□	□	□
Is a structured framework explicitly applied or analysed?	□	□	□
Does the study include evidence on preparedness, response, recovery or continuity?	□	□	□
Is the publication within the 2000–2025 timeframe?	□	□	□
Is the source peer-reviewed or credible grey literature?	□	□	□
Is the full text available in English?	□	□	□

Screening Decision: □ Include □ Exclude □ Full-text review

Reason for Exclusion (if applicable):

□ Not SME-focused

□ Not LMIC context

□ No relevant framework

□ Insufficient methodological detail

□ Not related to DRR/continuity

□ Other (specify): __________

## Appendix 3: Data Extraction Form

A structured template was used to extract and organise data from included studies to support synthesis and comparison.

**Table:**

Category	Data Extracted
Bibliographic information	
Geographical context	
Sector/industry	
SME characteristics	
Type of framework	
Framework application	
Implementation context	
Mechanisms	
Primary outcomes	
Secondary outcomes	
Measurement approach	
Study design	
Quality appraisal	
Key findings	
Barriers	
Enablers	
Policy implications	
Reviewer notes	
